# Carotenemia: A Case Report

**DOI:** 10.7759/cureus.5218

**Published:** 2019-07-23

**Authors:** Ehizogie Edigin, Iriagbonse R Asemota, Ezegwu Olisa, Chineme Nwaichi

**Affiliations:** 1 Internal Medicine, John H. Stroger Jr. Hospital of Cook County, Chicago, USA; 2 Internal Medicine, John H Stroger Jr. Hospital of Cook County, Chicago, USA

**Keywords:** carotenemia, jaundice, papaya, mango, lycopenemia

## Abstract

Carotenemia is a condition characterized by yellow-orange discoloration of the skin usually secondary to excessive ingestion of foods rich in carotene. It occurs in the absence of yellow discoloration of the sclera. Carotenemia is a benign condition; hence, further diagnostic testing is unnecessary. We present a case of carotenemia secondary to excessive ingestion of papaya and mango.

## Introduction

Carotenoderma is a phenomenon characterized by orange pigmentation of the skin, resulting from carotene deposition mainly in the stratum corneum. Different etiologies responsible for this phenomenon include high β-carotene intake from vegetables, fruits, eggs, and nutrient supplements and several metabolic states such as hypothyroidism, diabetes mellitus, pregnancy and anorexia nervosa, and familial carotenemia [1]. The absence of yellow pigment in the sclera and oral cavities distinguishes carotenemia from jaundice. Carotenemia is a benign condition. Awareness of carotenemia is essential to avoid confusion with jaundice and unnecessary diagnostic studies [2].

## Case presentation

A 43-year-old gentleman with a history of bronchial asthma and recently diagnosed distal gastric leiomyoma was admitted to the general surgical service for distal gastrectomy and Billroth I gastroduodenostomy.

He endorsed having chronic abdominal pain, heartburn, and poor oral intake for the past six months. He had noticed yellow-orange discoloration of both hands and feet for the past few weeks. He denied pruritus, darkening of the urine, and pale stools. Physical exam was significant for yellow-orange discolorations on the dorsal and ventral aspects of both hands, soles of both feet and axillae. His sclera was anicteric. Vital signs were within normal limits. Liver function test, coagulation profile, albumin, platelets, and thyroid function were within normal limits. On further interview, the patient reported that for the past six months, his diet has predominantly consisted of papaya and mango. The patient has been unable to tolerate other foods due to abdominal pain and heartburn secondary to his gastric mass. The patient was advised to cut down on foods rich in carotenoids, and pigmentation was expected to improve slowly over time.

The patient underwent planned surgery without any complication. He was discharged on the third postoperative day in stable condition. Patient reduced intake of carotenoid-rich foods, and yellow-orange skin dislocation was seen to be resolving on his one-month clinic follow up.

## Discussion

Carotenemia is a condition characterized by yellow discoloration of the skin and elevated blood carotene levels. Excessive and prolonged ingestion of carotene‐rich foods [3]. Fruits and vegetables rich in carotene include apple, orange, papaya, beans, peach, berries, pineapple, broccoli, brussels sprout, cabbage, pumpkin, carrot, spinach, squash, cucumber, tomato, lettuce, and mango. Other sources include butter, cheese, egg yolk, meat, milk, nutrient supplement, and palm oil [1]. Carotenoids are organic hydrocarbons mainly found in plant sources, and beta-carotene is the primary carotenoid found in plants. Beta-carotene is converted into vitamin A through two key enzymes, 15-15`-carotenoid dioxygenase and beta-carotene-15-15'-dioxygenase [4]. Carotene serves as the primary precursor of vitamin A in human. However, hypervitaminosis A does not occur with excess carotene ingestion as the body converts a limited quantity of carotene to vitamin A daily [5]. Carotenemia is common in infants and young children who have diets rich in green and orange vegetable purees [6]. Carotenemia tend to be limited to thick areas of the skin, such as the palms and soles, while Generalized carotenemia is rare [2].

**Figure 1:**
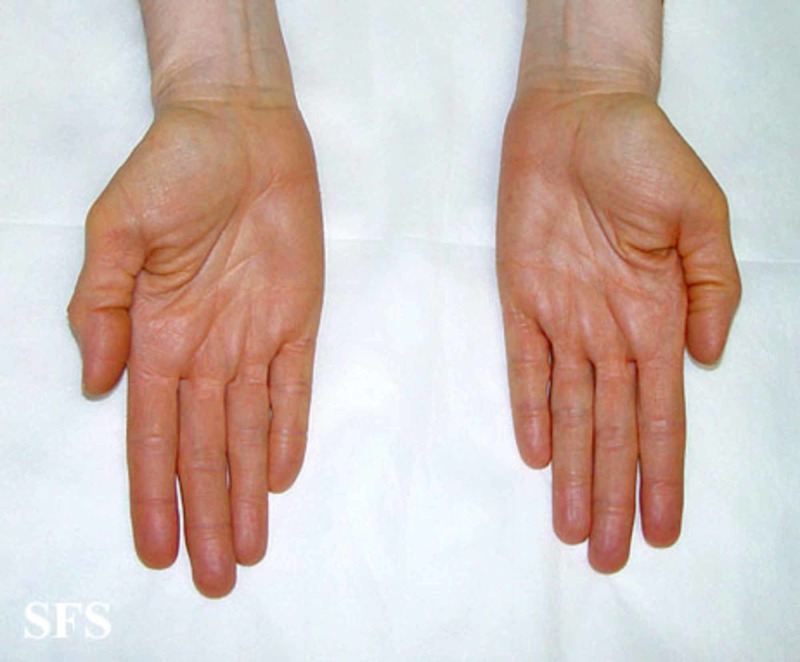
Carotenemia of the palms Picture from Dermatology Atlas; courtesy of Samuel Freire da Silva, M.D

**Figure 2:**
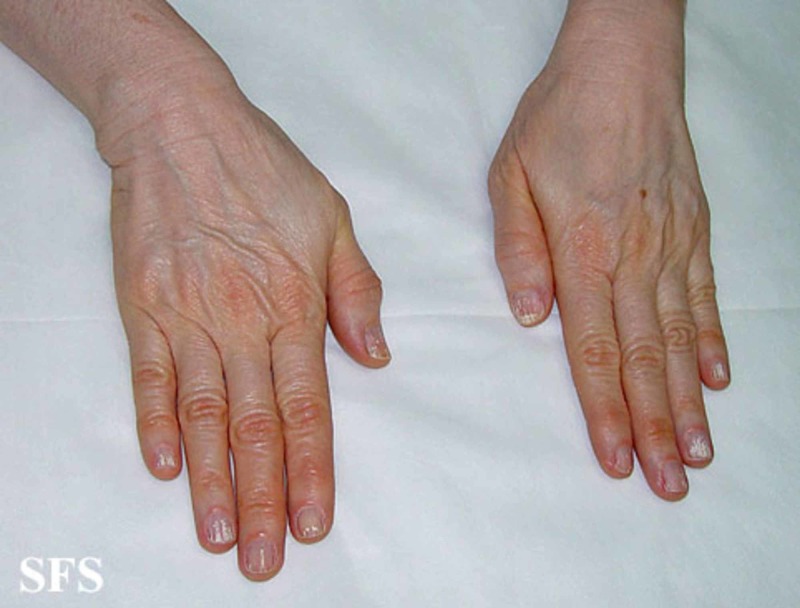
Carotenemia of the dorsum of the hands Picture from Dermatology Atlas; courtesy of Samuel Freire da Silva, M.D

Excessive dietary intake of carotene-rich food is by far the most common cause of carotenemia. In rare cases, it can result from systemic diseases like diabetes mellitus, nephritic syndrome, glomerulonephritis, hypothyroidism, anorexia nervosa, and primary hepatic disease. Carotenemia may also be related to restricted dietary habits, hyperlipidemia, a genetic defect in the metabolism of carotenoids in patients without a history of excessive carotene ingestion [7]. Evaluation of patients includes taking a detailed dietary history with a focus on the history of food consumption with high carotene with the estimation of the amount taken and duration. Yellow-orange skin pigmentation is the hallmark of the condition. Pigmentation spares the sclera and mucous membranes (unlike jaundice) and concentrates on the palms (Figure 1), dorsum of hands (Figure 2), soles, forehead, the tip of the nose and nasolabial folds [8]. A typical sign of carotenoderma is its enhanced appearance under artificial light [9]. Screening for systemic conditions that can present with carotenemia should be done.

Diet-induced carotenemia is a clinical diagnosis and laboratory confirmation is not generally required. However, it can be verified by a high serum beta carotene level, a normal or slightly elevated vitamin A level, and normal liver function test results [8]. The mainstay of treatment is reducing the amount of carotene in the diet, which will eventually lead to the resolution of skin pigmentation [10]. Reassurance should be provided to the patients and their families as this is a benign condition and unlikely to lead to any serious consequences. 

## Conclusions

Carotenemia most commonly result from excessive consumption of foods high in carotene. It is characterized by yellow-orange discoloration in the absence of yellow sclera and the presence of normal liver function test. It is a benign condition. Reduction in consumption of high carotene containing foods usually results in resolution of skin discoloration. It is essential for physicians to be aware of these condition in other to prevent unnecessary diagnostic testing for these patients.
